# Primary acantholytic squamous cell carcinoma of the cecum: a case report

**DOI:** 10.1186/1746-1596-6-5

**Published:** 2011-01-11

**Authors:** Zoran Jukić, Iva Ledinsky, Monika Ulamec, Mario Ledinsky, Božo Krušlin, Davor Tomas

**Affiliations:** 1 Department of Surgery, Nova Gradiska General Hospital, Nova Gradiska, Croatia; 2 Department of Surgery, Sestre milosrdnice University Hospital, Zagreb, Croatia; 3 Department of Pathology, Sestre milosrdnice University Hospital, Zagreb, Croatia; 4 School of medicine, University of Zagreb, Zagreb, Croatia

## Abstract

**Background:**

Acantholytic squamous cell carcinoma (ASCC) is an uncommon histopathologic variant of SCC, characterized by marked acantholysis, wherein the tumor cells demonstrate defective cohesion to one another in the cancer nest leading to a pseudoglandular or pseudovascular appearance. The most common site of ASCC is the sun-exposed areas of the skin. Sporadic cases of ASCC have also been reported in various mucosal membranes and organs but to our knowledge this is the first case of primary ASCC of the large bowel.

**Case presentation:**

A 59-year-old woman underwent right hemicolectomy due to large tumor in cecum and initial part of the ascending colon. Microscopically, the tumor consisted of nests of focally keratinizing large, atypical, squamous epithelial cells. Approximately 70% of the tumor showed acantholytic changes and acantholysis was equally distributed through the entire tumor. Immunohistochemically tumor cells were diffusely positive for cytokeratin (CK) AE1/AE3 and focally positive for epithelial membrane antigen and syndecan 1. All other tested antibodies (CK7, CK 20, CK MNF116, E-cadherin, beta-catenin, p63, p16, CD31, CD34, CEA, estrogen, progesterone) showed negative reaction. Periodic acid Schiff and alcian blue staining showed no intracellular or extracellular mucinous material in the tumor. The diagnosis of acantholytic squamous cell carcinoma of the cecum was suspected and additional examination was recommended to exclude possibility of metastatic carcinoma. Extensive clinical examination which also included whole-body PET/CT scan showed no additional tumors. After the exclusion of possible metastatic disease the diagnosis of primary acantholytic squamous cell carcinoma of the cecum was confirmed. Six months after surgery the metastasis in small intestine and recurrence in the abdominal cavity at the site of surgery appeared and had the same morphological characteristic as the primary tumor in the cecum.

**Conclusion:**

We report a unique case of ASCC arising in cecum and on this way expands the range of tumors originating in colon. Reports of more cases of colonic ASCC would possibly help to elucidate origin, clinical behavior and therapy of these tumors.

## Background

Primary squamous cell carcinoma (SCC) of the cecum is rare entity and only limited numbers of cases are described [1-4]. The first case of SCC was reported by Schmidtman in 1919 [5]. Acantholytic squamous cell carcinoma (ASCC) is an uncommon histopathologic variant of SCC, characterized by marked acantholysis, wherein the tumor cells demonstrate defective cohesion to one another in the cancer nest leading to a pseudoglandular or pseudovascular appearance [6]. It was first described in details by Lever [7] in 1947 as adenoacanthoma of the sweat glands. Synonyms include adenoid SCC, pseudoglandular SCC, SCC with gland-like features, angiosarcoma-like SCC, and pseudovascular adenoid SCC. The most common site of ASCC is the sun-exposed areas of the skin, particularly on the head and neck of elderly men [6]. Sporadic cases of ASCC have also been reported in various mucosal membranes and organs [8-12] but to our knowledge this is the first case of primary ASCC of the large bowel.

## Case presentation

A 59-year-old woman who presented with a one month history of lower abdominal pain and intermittent watery, bloody stool was admitted to our hospital. On physical examination, she was found to have right lower quadrant abdominal tenderness without guarding. Blood tests revealed anemia and C reactive protein was slightly elevated. Her past medical history included hysterectomy due to multiple myomas 25 years earlier and segmentectomy of right breast 3 years ago. Patohistological diagnosis of breast lesion was fibrocystic breast disease with usual and atypical intraductal epithelial proliferation and foci of adenosis without sign of intraductal or invasive carcinoma. Her mother and brother died from conventional colon cancer in age of 69 and 63 years, respectively. Father died of small cell lung cancer in the age of 67 years. Colonoscopy was performed and showed large tumor in cecum and initial part of the ascending colon. A biopsy confirmed malignant nature of the process and diagnosis of squamous cell carcinoma was suggested. Abdominal CT showed large tumor in cecoascending region of the colon and enlarged regional lymph nodes. Signs of distant metastasis are not found. Chest X-ray was unremarkable. A right hemicolectomy with removal of adjacent pericolic lymph nodes was carried out.

**Figure 1:**
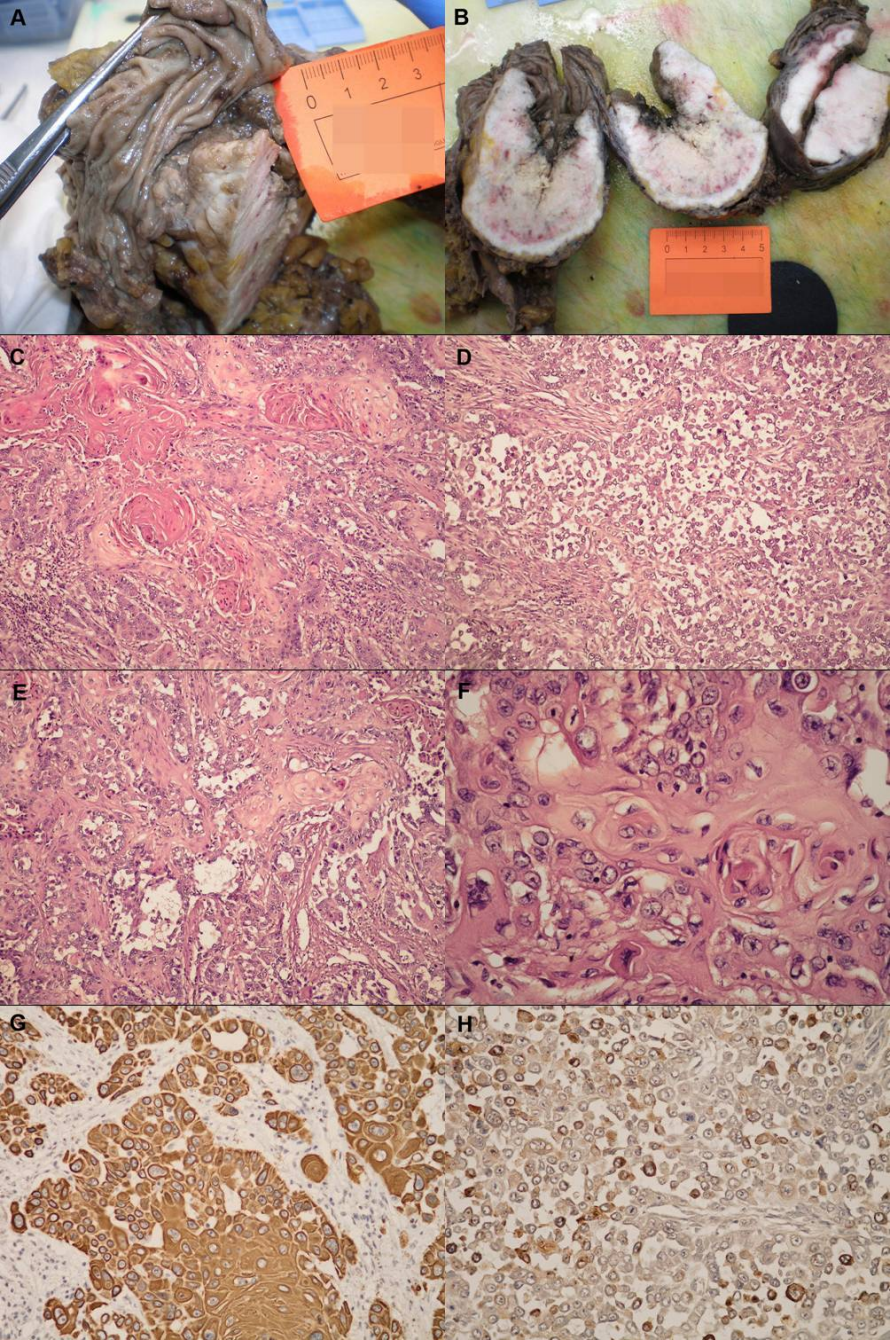
**Macroscopic, microscopic and immunohistochemical features of the acantholytic squamous cell carcinoma of the cecum.** (A) Grossly, in the cecoascending region, an ulcero-proliferative, whitish-gray tumor was found. (B) The tumor infiltrated all layers of the bowel wall and spread deeply into the surrounding fatty tissue. (C) Microscopically, the tumor consisted of large, atypical, squamous epithelial cells with abundant keratin formation (H&E, × 100). (D) Large areas of tumor showed prominent acantholythic changes (H&E, × 100). (E) Pseudoglandular formation with detached keratinocytes and inflammatory cells were also detected (H&E, × 100). (F) Tumor cells were large with bright eosinophilic, focally glassy cytoplasm and vesicular nuclei with small to medium sized nucleoli (400×). (G) Immunohistochemically tumor cells were diffusely positive for cytokeratin AE1/AE3 including acantholytic and pseudoglandular area (× 200). (H) Syndecan-1 expression was reduced and confined on small group of detached malignant cell (× 200).

Grossly, in the cecoascending region, an ulcero-proliferative, whitish-gray tumor measuring 9 cm in maximum diameter was found (Figure 1A and 1B). Microscopically, the tumor consisted of nests of focally keratinizing, atypical, squamous epithelial cells (Figure 1C). Approximately 70% of the tumor showed acantholytic changes and acantholysis was equally distributed through the entire tumor (Figure 1D). In the lumen-like pseudoglandular spaces, detached keratinocytes and inflammatory cell were detected (Figure 1E). Tumor cells were large with bright eosinophilic, focally glassy cytoplasm and vesicular nuclei with small to medium sized nucleoli (Figure 1F). The spindle cell carcinoma elements were not found. The tumor infiltrated all layers of the bowel wall and spread deeply into the surrounding fatty tissue. Twenty-two lymph nodes received with the specimen did not show any metastases.

Immunohistochemical staining was performed following the microwave streptavidin immunoperoxidase (MSIP) protocol on DAKO TechMate Horizon automated immunostainer using the following primary antibodies: cytokeratin (CK) AE1/AE3, CK7, CK 20, CK MNF116, EMA, E-cadherin, beta-catenin, p63, p16, CD31, CD34, CEA, estrogen, progesterone (all antibodies were purchased from DAKO, Denmark). Appropriate positive and negative control was used for each antibody. Periodic acid Schiff and alcian blue staining were made following the standard procedure.

**Figure 2:**
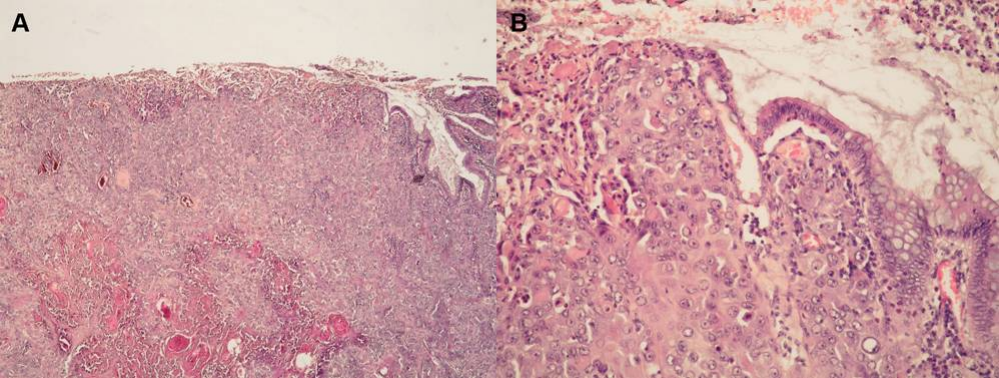
**The tumor-cecum mucosa boundary of the acantholytic squamous cell carcinoma of the cecum.** (A) The tumor began in the colonic mucosa and was top heavy (H&E, × 40). (B) The tumor-cecum mucosa boundary also indicated primary tumor (H&E, × 200).

Immunohistochemically tumor cells were diffusely positive for CK AE1/AE3 (Figure 1G) and focally positive for epithelial membrane antigen and syndecan 1 (Figure 1H). All other tested antibodies showed negative reaction. Periodic acid Schiff and alcian blue staining showed no intracellular or extracellular mucinous material in the tumor, including acantholytic and pseudoglandular areas. The microscopic features of the tumor-cecum mucosa boundary indicated primary tumor (Figure 2A and 2B) and the diagnosis of acantholytic squamous cell carcinoma of the cecum were suspected. Additional extensive examination was recommended to exclude possibility of metastatic carcinoma. The patient underwent gynecological, head and neck and pulmological examination. Clinical examination and pelvic and breast ultrasound, mammography, head and neck and lung MRI found no additional tumor. Finally, the patient underwent whole-body PET/CT scan which also showed no additional tumors. After the exclusion of possible metastatic disease the diagnosis of primary acantholytic squamous cell carcinoma of the cecum was confirmed.

The patient had no complications during the postoperative course and was discharged 12 days after surgery with suggested oncological examination and treatment. Additional chemotherapy was performed and the patients received 5-florouracil and folinic acid during six months. Radiation therapy was not applied. Six months after surgery the metastasis in small intestine and recurrence in the abdominal cavity at the site of surgery appeared and had the same morphological characteristic as the primary tumor in the cecum.

## Discussion

In ASCC of the skin and breast different molecular alterations have been associated with loss of cellular adhesion, including loss of E-cadherin or syndecan-1 [12,13]. In our case tumor cell showed loss of E-cadherin and beta-catenin while syndecan-1 expression was reduced and confined on small group of detached malignant cell. Loss of E-cadherin, beta-catenin and altered syndecan-1 expression suggest an impaired cellular adhesion, which may explain the peculiar histological appearance of these tumors and contribute to the observed aggressive clinical behavior in skin, penile and breast ASCC [6,10,12]. In our case tumor had also aggressive course but making conclusions about clinical behavior of this type tumor in colon are difficult because this is a first case described with limited follow-up.

Before regarding such a case as primary SCC of the colon, especially when tumor is located 7 cm proximal to the dentate line, exclusion of possibilities of metastatic deposit from a source elsewhere or direct extension from other site is necessary [1,14]. In addition, the affected segment of bowel could not be in continuity with squamous lined fistula or the anal squamous epithelium, mucin should be absent, intercellular bridges should be visible and keratin may or may not be present [1,14]. Some predisposing conditions are thought to play a role in the development of primary colonic SCC such as inflammatory bowel disease (especially ulcerative colitis), schistostomiasis, pelvic irradiation, villous adenoma and duplication of the intestine [1-4,14]. Our patient had no any mentioned predisposing conditions but her family history could suggest some genetic alterations that may have role in tumor formation and especially suspected was Lynch syndrome. Lynch syndrome or hereditary non-polyposis colorectal cancer (HNPCC) is an autosomal dominant genetic condition which is characterized with a high risk of colon cancer as well as other cancers including endometrium, ovary, stomach, small intestine, hepatobiliary tract, upper urinary tract, brain, and skin. The increased risk for these cancers is due to inherited mutations that impair DNA mismatch repair. Patients with Lynch syndrome usually present with adenocarcinoma of the proximal colon (cecum), with or without synchronous or metachronous colorectal cancer or other malignancy typical of the syndrome. The majority of colorectal carcinomas arising in Lynch syndrome are generally similar to sporadic colorectal cancer but mucinous, signet-ring and medullary types are identified with increased frequency in Lynch syndrome patients [15]. In addition, the presence of tumor-infiltrating lymphocytes and especially presence of intraepithelial T-lymphocytes could identify the majority of colorectal cancers with this mutation [16]. Despite patient denied additional cancers in family and did not know type and localization of colorectal cancer in relatives we recommended additional genetic testing to exclude possibility of Lynch syndrome.

We also considered HPV as potential carcinogen but immunostaining for p16 was negative and therefore additional investigations were not performed.

It is important for pathologist to discriminate between primary colonic ASCC and metastasis of any SCC or ASCC from elsewhere. Likewise, metastasis of endometroid carcinoma with squamous differentiation or transitional cell carcinoma with gland formation and squamous differentiation should be taken into consideration. Endometroid carcinomas are immunohistochemically positive for CK7, progesterone and estrogen receptors while transitional cell carcinomas are positive for CK7 and CK20. In our case these markers were negative and CK AE1/AE3 was strongly and diffusely positive in whole tumor including pseudoglandular and acantholytic areas. Recommended additional extensive clinical examination and immunohistochemical analysis excluded possible metastatic disease and patient fulfils all criteria for diagnosis of primary ASCC carcinoma.

There are four main proposed pathogenic theories trying to explain the origin of SCC of the colon [1,2]. Squamous cells which are the source for SCC could originate from proliferation of uncommitted reserve or basal cells following mucosal injury, from squamous metaplasia of glandular epithelium resulting from chronic irritation, from embryonal nests of ectodermal cells or from stem cells [1,2].

Colonic ASCC should be also differentiated from adenosquamous carcinoma and angiosarcoma. Adenosquamous carcinoma is defined as a tumor with malignant glandular and squamous component and the potential for metastases [17]. In the colon and rectum, this tumor is extremely rare and accounts for 0.1% to 0.2% of all colon cancers. Histogenesis of adenosquamous carcinoma is unclear and several theories, that are similar to theories of origin of colonic SCC, have been proposed to try to explain squamous differentiation in colonic mucosa [17]. Malignant glandular component in adenosquamous colonic carcinoma retain immunohistochemical characteristic of conventional colonic adenocarcinoma (positivity for CK20 and CEA) and contain mucinous material in the lumen or cells cytoplasms [17].

Another rare tumor to be distinguished from ASCC is the angiosarcoma, especially in the variant of ASCC termed pseudovascular or angiosarcoma-like SCC where anastomosing spaces and channels that are mimicking neoplastic vessels of angiosarcoma are formed and keratin formation is absent [8,9]. Angiosarcoma occurs very rarely in the intestinal tract as either primary or metastatic malignancy and can present great diagnostic difficulty, especially when it displays epithelioid cytomorphology [18]. An incorrect diagnosis of angiosarcoma may lead to inappropriate treatment and prognosis. Immunohistochemical examination is a useful tool in making the distinction but cytokeratins and epithelial membrane antigen should not be considered to be a distinct discriminating criterion, because malignant endothelial cells may be positive [18]. Allison et al. [18] immunohistochemically analyzed a series of 8 primary and metastatic angiosarcoma involving the gastrointestinal tract and found that tumor cells were immunoreactive for cytokeratins AE1/AE3 (7/8), cytokeratin 7 (2/8), Cam5.2/cytokeratin 8 (5/8), and cytokeratin 19 (5/8). In one case weakly and focal positivity for epithelial membrane antigen was also observed [18]. In contrast to an angiosarcoma, the cells of ASCC are negative for endothelial markers such as CD31, CD34 and von Willebrand factor and these markers must be included in differentiating ASCC from angiosarcoma [8,9,18].

The immunohistochemical and histochemical findings of the present tumor were compatible with the previous data of ASCC and exclude possibility of adenosquamous carcinoma or angiosarcoma [8-13].

## Conclusion

We report a unique case of ASCC arising in cecum and on this way expands the range of tumors originating in colon. Reports of more cases of colonic ASCC would possibly help to elucidate origin, clinical behavior and therapy of these tumors.

## Consent

Written informed consent was obtained from the patient for publication of this case report and accompanying images. A copy of the written consent is available for review by the Editor-in-Chief of this journal.

## Competing interests

The authors declare that they have no competing interests.

## Authors' contributions

ZJ supplied relevant clinical information about the patient, drafted the manuscript and was involved in manuscript revision. IL and ML were involved in literature search and preparing the material. MU acquired photomicrographs and drafted the manuscript. BK participated in the histopathological evaluation and drafted the manuscript. DT participated in the histopathological evaluation, outlined the general concept of the manuscript, and has been involved in drafting and revising it critically. All authors have read and approved the final manuscript.
